# (±)-Cyclo­hexane-1,2-diyl bis­(4-nitro­benzoate)

**DOI:** 10.1107/S1600536808033874

**Published:** 2008-10-22

**Authors:** Sok Teng Tong, David Barker, Ka Wai Choi, Peter D. W. Boyd, Margaret A. Brimble

**Affiliations:** a Department of Chemistry, University of Auckland, Private Bag 92019, Auckland, New Zealand

## Abstract

The crystal structure of the title compound, C_20_H_18_N_2_O_8_, has been investigated to establish the relative stereochemistry between the ester groups. The cyclo­hexane ring adopts a chair conformation, in which the two ester groups occupy the adjacent equatorial positions in a *trans* relationship with each other. The mol­ecules assemble in the crystal as chains along the *c* axis *via* C—H⋯π inter­actions between the cyclo­hexane ring and a pair of nitro­phenyl rings of the neighbouring mol­ecule. Also observed are π–π stacking inter­actions between the nitro­phenyl rings of neighbouring chains, with a perpendicular distance between these rings of 3.409 Å and a slippage of 0.969 Å.

## Related literature

For the related synthesis of cyclo­hexane-1,2-diyl-bis­(4-bromo­benzoate) from *trans*-cyclo­hexane-1,2-diol, see: Hayashi *et al.* (2004 ▶); for non-conventional hydrogen contacts and stacking inter­actions, see: Desiraju & Steiner (2001 ▶) and Ciunik & Jarosz (1998 ▶).
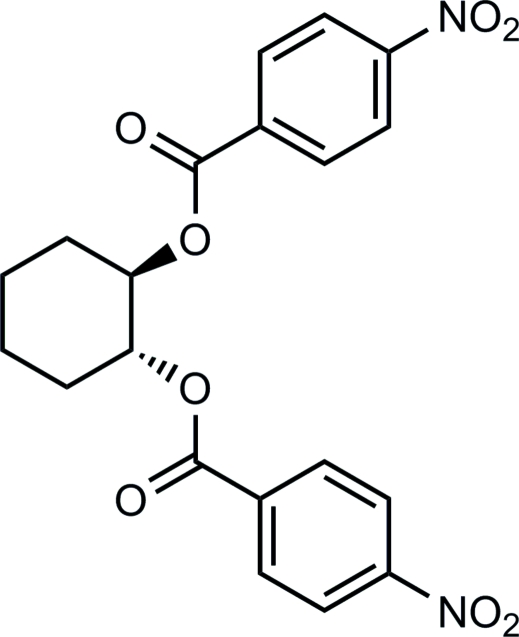


## Experimental

### 

#### Crystal data

C_20_H_18_N_2_O_8_
*M* *_r_* = 414.36
Monoclinic, 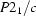

*a* = 12.6510 (2) Å
*b* = 12.2720 (2) Å
*c* = 13.2186 (2) Å
β = 108.8300 (10)°
*V* = 1942.39 (5) Å^3^
*Z* = 4
Mo *K*α radiation
μ = 0.11 mm^−1^
*T* = 89 (2) K
0.2 × 0.1 × 0.05 mm

#### Data collection

Siemens SMART diffractometer with an APEXII CCD detector
Absorption correction: none
15207 measured reflections
4973 independent reflections
2999 reflections with *I* > 2σ(*I*)
*R*_int_ = 0.077

#### Refinement

*R*[*F* ^2^ > 2σ(*F* ^2^)] = 0.041
*wR*(*F* ^2^) = 0.098
*S* = 0.91
4973 reflections
271 parameters
H-atom parameters constrained
Δρ_max_ = 0.28 e Å^−3^
Δρ_min_ = −0.30 e Å^−3^

### 

Data collection: *SMART* (Siemens, 1995 ▶); cell refinement: *SAINT* (Siemens, 1995 ▶); data reduction: *SAINT*; program(s) used to solve structure: *SHELXS97* (Sheldrick, 2008 ▶); program(s) used to refine structure: *SHELXL97* (Sheldrick, 2008 ▶); molecular graphics: *ORTEPIII* (Burnett & Johnson, 1996 ▶) and *Mercury* (Macrae *et al.*, 2006 ▶); software used to prepare material for publication: *WinGX* (Farrugia, 1999 ▶) and *publCIF* (Westrip, 2008 ▶).

## Supplementary Material

Crystal structure: contains datablocks I, global. DOI: 10.1107/S1600536808033874/si2117sup1.cif

Structure factors: contains datablocks I. DOI: 10.1107/S1600536808033874/si2117Isup2.hkl

Additional supplementary materials:  crystallographic information; 3D view; checkCIF report

## Figures and Tables

**Table 1:** Hydrogen-bond geometry (Å, °)

*D*—H⋯*A*	*D*—H	H⋯*A*	*D*⋯*A*	*D*—H⋯*A*
C14—H14⋯O8^i^	0.93	2.40	3.240 (2)	150
C16—H16⋯O3	0.93	2.57	3.4374 (19)	156
C19—H19⋯O3^ii^	0.93	2.29	3.0919 (19)	145
C5—H5*A*⋯*Cg*2^iii^	0.97	2.79	3.7273 (18)	162

Symmetry codes: (i) 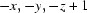; (ii) 
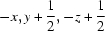
; (iii) 
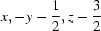
.

**Fig. 1.**
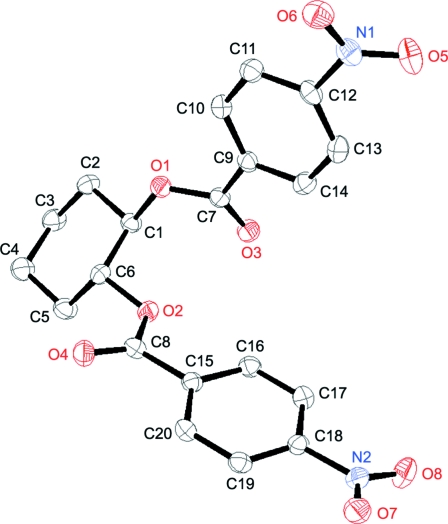
The molecular structure and atom numbering scheme of (1R*,2R*)-diester (1R*,6R* in the crystallographic numbering scheme) with displacement ellipsoids drawn at the 50% probability level for non-H atoms.

**Fig. 2.**
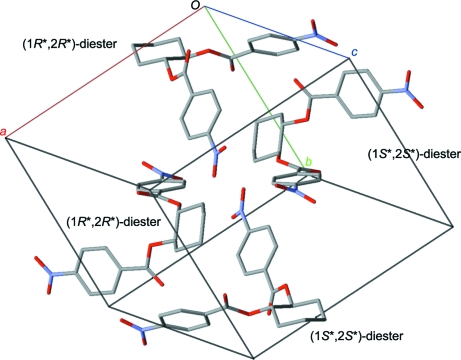
The molecular packing of racemic diester withing a unit cell. The origin of the unit cell is labelled as O while cell axes are labelled as a (red), b (green) and c (blue), respectively.

**Fig. 3.**
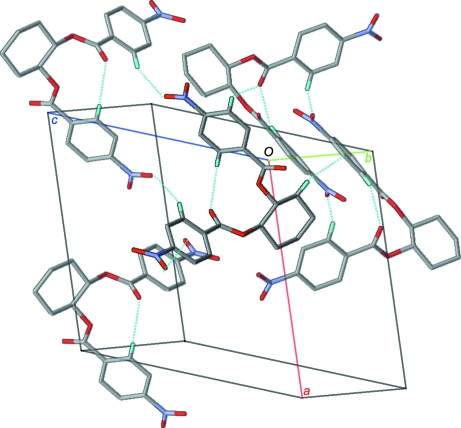
The molecular packing of racemic diester with non-conventional hydrogen bonding represented as dashed lines. Hydrogen atoms not involved in hydrogen bonding have been omitted for clarity.

## supplementary crystallographic information

### Comment

The title diester was isolated as a part of the study towards the organocatalytic *α*-oxidation of cyclohexanone catalysed by (*S*)-proline. Using 3-phenyl-2-tosyl-1,2-oxaziridine as the oxidant, cyclohexanone was oxidized to *α*-hydroxycyclohexanone which was subsequently reduced *in-situ* to the corresponding diol with sodium borohydride. Concomitant esterification of the two hydroxyl groups afforded the title diester.

The crystal structure reveals the relative stereochemistry of the racemic diester to be 1*R**,2*R** and 1*S**,2*S** (1*R**,6*R** and 1*S**,6*S** in the crystallographic numbering scheme, Fig. 1). The two adjacent ester groups occupy the *trans* diequatorial position of the cyclohexane ring in the chair conformation with a C_2_ rotational axis bissecting the cyclohexane ring between the two ester groups (Figure 1). The cyclohexane moiety fits into a cleft formed by the two nitrophenyl groups of a neighbouring diester of opposite stereochemistry with an interplanar angle of 89.16 (6)° (Fig. 2) and the molecules are connected to each other *via* C—H···π interactions and C—H···O contacts (Table 1). Weak non-conventional C—H···O and C—H···π contacts are extensively discussed by Desiraju & Steiner (2001), and a combination of C—H···O and π···π stacking interactions are reported by Ciunik & Jarosz (1998).These interactions lead to the formation of chains of molecules running along the *c* axis. Each chain is surrounded by four similar chains that are propagated in the opposite direction, and π···π stacking interactions are observed between the nitrophenyl groups of neighbouring chains (Fig. 3). The centroid (Cg) separation between the stacking phenyl ring (*Cg*2, C9–C14) and the neighbouring ring (*Cg*2^iv^), is 3.5441 (9) Å, (symmetry code iv = 1 - *x*, - *y*, 1 - *z*). The slippage of the two rings is 0.969 Å along the 1,4 (C9–C12) vector and the perpendicular distance between *Cg*2 and the second symmetry related ring is 3.409 Å.

### Experimental

To a solution of cyclohexane-1,2-diol (19.0 mg, 0.164 mmol) in CH_2_Cl_2_ (1.2 ml) was added triethylamine (0.400 ml, 2.89 mmol), 4-nitrobenzoyl chloride (152 mg, 0.818 mmol) and a catalytic amount of 4-(dimethylamino)-pyridine at room temperature. After overnight stirring, the mixture was quenched with pH 7 phosphate buffer (2 ml). The organic phase was separated and the aqueous phase was extracted with EtOAc (5 ml *x* 3). The combined organic extracts were washed with brine, dried over MgSO_4_ and concentrated *in vacuo* to afford a crude dark brown oil. Purification by flash chromatography using hexane-EtOAc (4:1) as eluent furnished the title diester as a brown solid (64.0 mg, 94%). Recrystallization of the title diester from methanol afforded yellow needles. Melting point: 381 K.

### Refinement

H atoms were placed in calculated positions and were refined using a riding model (C–H = 0.93 or 0.97 Å), with *U*_iso_(H) = 1.2 or 1.5 times *U*_eq_(C).

### Crystal data

**Table:**

C_20_H_18_N_2_O_8_	*D*_x_ = 1.417 Mg m^−^^3^
*M**_r_* = 414.36	Melting point: 381(1) K
Monoclinic, *P*2_1_/*c*	Mo *K*α radiation, λ = 0.71073 Å
*a* = 12.6510 (2) Å	Cell parameters from 4298 reflections
*b* = 12.2720 (2) Å	θ = 1.7–25.1°
*c* = 13.2186 (2) Å	µ = 0.11 mm^−^^1^
β = 108.830 (1)°	*T* = 89 K
*V* = 1942.39 (5) Å^3^	Needle, yellow
*Z* = 4	0.2 × 0.1 × 0.05 mm
*F*(000) = 864	

### Data collection

**Table:**

Siemens SMART diffractometer with an APEXII CCD detector	2999 reflections with *I* > 2σ(*I*)
Radiation source: fine-focus sealed tube	*R*_int_ = 0.077
graphite	θ_max_ = 28.8°, θ_min_ = 1.7°
area–detector ω scans	*h* = −16→17
15207 measured reflections	*k* = −16→16
4973 independent reflections	*l* = −17→17

### Refinement

**Table:**

Refinement on *F*^2^	Primary atom site location: structure-invariant direct methods
Least-squares matrix: full	Secondary atom site location: difference Fourier map
*R*[*F*^2^ > 2σ(*F*^2^)] = 0.041	Hydrogen site location: inferred from neighbouring sites
*wR*(*F*^2^) = 0.098	H-atom parameters constrained
*S* = 0.91	*w* = 1/[σ^2^(*F*_o_^2^) + (0.0453*P*)^2^] where *P* = (*F*_o_^2^ + 2*F*_c_^2^)/3
4973 reflections	(Δ/σ)_max_ < 0.001
271 parameters	Δρ_max_ = 0.28 e Å^−^^3^
0 restraints	Δρ_min_ = −0.30 e Å^−^^3^

### Special details

**Table:**

Geometry. All e.s.d.'s (except the e.s.d. in the dihedral angle between two l.s. planes) are estimated using the full covariance matrix. The cell e.s.d.'s are taken into account individually in the estimation of e.s.d.'s in distances, angles and torsion angles; correlations between e.s.d.'s in cell parameters are only used when they are defined by crystal symmetry. An approximate (isotropic) treatment of cell e.s.d.'s is used for estimating e.s.d.'s involving l.s. planes.
Refinement. Refinement of *F*^2^ against ALL reflections. The weighted *R*-factor *wR* and goodness of fit *S* are based on *F*^2^, conventional *R*-factors *R* are based on *F*, with *F* set to zero for negative *F*^2^. The threshold expression of *F*^2^ > σ(*F*^2^) is used only for calculating *R*-factors(gt) *etc*. and is not relevant to the choice of reflections for refinement. *R*-factors based on *F*^2^ are statistically about twice as large as those based on *F*, and *R*- factors based on ALL data will be even larger.

### Fractional atomic coordinates and isotropic or equivalent isotropic displacement parameters (Å^2^)

**Table:**

	*x*	*y*	*z*	*U*_iso_*/*U*_eq_	
O2	0.13143 (8)	0.12316 (9)	0.12240 (8)	0.0237 (2)	
O4	0.07109 (9)	0.29575 (9)	0.08144 (8)	0.0294 (3)	
O1	0.36109 (8)	0.08691 (8)	0.21140 (7)	0.0238 (3)	
O3	0.26940 (8)	−0.03067 (9)	0.28553 (8)	0.0263 (3)	
O6	0.62313 (9)	0.29785 (10)	0.71389 (8)	0.0337 (3)	
O7	−0.20229 (9)	0.24599 (10)	0.45414 (8)	0.0341 (3)	
O5	0.55152 (10)	0.16681 (9)	0.78095 (8)	0.0368 (3)	
O8	−0.16503 (10)	0.07314 (10)	0.46935 (9)	0.0371 (3)	
C9	0.39653 (12)	0.09399 (12)	0.39838 (11)	0.0213 (3)	
N1	0.56407 (11)	0.21667 (11)	0.70528 (10)	0.0280 (3)	
N2	−0.16168 (10)	0.16318 (12)	0.42995 (10)	0.0269 (3)	
C18	−0.10386 (12)	0.17208 (13)	0.34967 (11)	0.0221 (3)	
C1	0.29657 (12)	0.04900 (13)	0.10370 (11)	0.0224 (3)	
H1	0.2646	−0.0229	0.1080	0.027*	
C12	0.50482 (13)	0.17579 (12)	0.59718 (11)	0.0236 (3)	
C6	0.20401 (12)	0.13022 (13)	0.05672 (11)	0.0235 (3)	
H6	0.2354	0.2038	0.0614	0.028*	
C15	0.00784 (12)	0.19370 (13)	0.20538 (10)	0.0206 (3)	
C16	0.02556 (12)	0.10200 (13)	0.27072 (11)	0.0238 (4)	
H16	0.0757	0.0487	0.2655	0.029*	
C14	0.35143 (13)	0.07916 (13)	0.48064 (12)	0.0261 (4)	
H14	0.2843	0.0420	0.4676	0.031*	
C17	−0.03163 (12)	0.09018 (13)	0.34374 (11)	0.0247 (4)	
H17	−0.0215	0.0289	0.3873	0.030*	
C10	0.49575 (13)	0.15077 (13)	0.41674 (11)	0.0240 (3)	
H10	0.5254	0.1603	0.3615	0.029*	
C20	−0.06729 (12)	0.27323 (13)	0.21196 (11)	0.0226 (3)	
H20	−0.0799	0.3335	0.1669	0.027*	
C13	0.40625 (13)	0.11952 (13)	0.58144 (12)	0.0270 (4)	
H13	0.3776	0.1091	0.6373	0.032*	
C4	0.21805 (13)	0.09860 (14)	−0.12498 (12)	0.0315 (4)	
H4A	0.1763	0.0791	−0.1981	0.038*	
H4B	0.2514	0.1697	−0.1257	0.038*	
C11	0.55089 (13)	0.19336 (13)	0.51698 (12)	0.0254 (4)	
H11	0.6169	0.2326	0.5299	0.031*	
C7	0.33501 (12)	0.04317 (13)	0.29358 (12)	0.0225 (3)	
C5	0.13918 (13)	0.10373 (15)	−0.05883 (11)	0.0302 (4)	
H5A	0.0829	0.1592	−0.0878	0.036*	
H5B	0.1016	0.0342	−0.0625	0.036*	
C8	0.07180 (12)	0.21209 (13)	0.12914 (11)	0.0226 (3)	
C19	−0.12367 (12)	0.26323 (13)	0.28548 (11)	0.0226 (3)	
H19	−0.1735	0.3166	0.2914	0.027*	
C3	0.30937 (13)	0.01510 (14)	−0.07859 (12)	0.0297 (4)	
H3A	0.3599	0.0142	−0.1203	0.036*	
H3B	0.2763	−0.0568	−0.0826	0.036*	
C2	0.37471 (12)	0.04183 (14)	0.03779 (11)	0.0259 (4)	
H2A	0.4302	−0.0143	0.0670	0.031*	
H2B	0.4134	0.1107	0.0412	0.031*	

### Atomic displacement parameters (Å^2^)

**Table:**

	*U*^11^	*U*^22^	*U*^33^	*U*^12^	*U*^13^	*U*^23^
O2	0.0232 (5)	0.0264 (6)	0.0246 (5)	0.0030 (5)	0.0120 (4)	0.0016 (5)
O4	0.0333 (6)	0.0289 (6)	0.0296 (6)	0.0057 (6)	0.0154 (5)	0.0067 (5)
O1	0.0238 (6)	0.0269 (6)	0.0198 (5)	−0.0028 (5)	0.0057 (4)	−0.0001 (5)
O3	0.0266 (6)	0.0235 (6)	0.0279 (6)	−0.0040 (5)	0.0077 (5)	−0.0001 (5)
O6	0.0331 (6)	0.0315 (7)	0.0330 (6)	−0.0044 (6)	0.0057 (5)	−0.0059 (6)
O7	0.0320 (6)	0.0451 (8)	0.0290 (6)	0.0127 (6)	0.0150 (5)	0.0018 (6)
O5	0.0551 (8)	0.0320 (7)	0.0227 (6)	0.0051 (6)	0.0118 (5)	0.0032 (5)
O8	0.0417 (7)	0.0364 (7)	0.0406 (7)	−0.0056 (6)	0.0236 (6)	0.0053 (6)
C9	0.0220 (8)	0.0188 (8)	0.0218 (7)	0.0018 (7)	0.0053 (6)	0.0023 (7)
N1	0.0317 (8)	0.0258 (8)	0.0247 (7)	0.0069 (7)	0.0065 (6)	−0.0003 (6)
N2	0.0208 (7)	0.0354 (9)	0.0248 (7)	−0.0002 (7)	0.0078 (6)	0.0009 (7)
C18	0.0185 (7)	0.0299 (9)	0.0183 (7)	0.0004 (7)	0.0066 (6)	−0.0008 (7)
C1	0.0207 (8)	0.0255 (9)	0.0197 (7)	−0.0011 (7)	0.0046 (6)	−0.0016 (7)
C12	0.0279 (8)	0.0198 (8)	0.0205 (7)	0.0033 (7)	0.0041 (6)	0.0003 (7)
C6	0.0221 (8)	0.0273 (9)	0.0240 (8)	0.0009 (7)	0.0114 (6)	0.0018 (7)
C15	0.0190 (7)	0.0251 (8)	0.0165 (7)	−0.0001 (7)	0.0043 (6)	−0.0020 (7)
C16	0.0224 (8)	0.0255 (9)	0.0245 (8)	0.0056 (7)	0.0087 (6)	0.0006 (7)
C14	0.0242 (8)	0.0257 (9)	0.0292 (8)	−0.0031 (8)	0.0098 (7)	0.0003 (7)
C17	0.0260 (8)	0.0242 (9)	0.0235 (8)	0.0009 (7)	0.0075 (6)	0.0030 (7)
C10	0.0268 (8)	0.0232 (9)	0.0225 (7)	0.0002 (8)	0.0087 (6)	0.0025 (7)
C20	0.0230 (8)	0.0237 (9)	0.0189 (7)	0.0018 (7)	0.0038 (6)	0.0014 (7)
C13	0.0336 (9)	0.0265 (9)	0.0245 (8)	0.0009 (8)	0.0142 (7)	0.0014 (7)
C4	0.0302 (9)	0.0416 (11)	0.0220 (8)	0.0001 (9)	0.0077 (7)	−0.0028 (8)
C11	0.0249 (8)	0.0234 (9)	0.0271 (8)	−0.0015 (7)	0.0071 (7)	0.0002 (7)
C7	0.0205 (8)	0.0217 (8)	0.0251 (8)	0.0032 (7)	0.0072 (6)	0.0033 (7)
C5	0.0248 (8)	0.0420 (11)	0.0233 (8)	0.0040 (8)	0.0069 (7)	−0.0009 (8)
C8	0.0219 (8)	0.0238 (9)	0.0204 (7)	0.0026 (7)	0.0044 (6)	−0.0005 (7)
C19	0.0203 (8)	0.0248 (9)	0.0221 (7)	0.0041 (7)	0.0057 (6)	−0.0013 (7)
C3	0.0283 (9)	0.0353 (10)	0.0277 (8)	−0.0006 (8)	0.0122 (7)	−0.0057 (8)
C2	0.0214 (8)	0.0294 (9)	0.0285 (8)	−0.0004 (8)	0.0101 (7)	−0.0018 (7)

### Geometric parameters (Å, °)

**Table:**

O2—C8	1.3459 (18)	C15—C16	1.392 (2)
O2—C6	1.4557 (16)	C15—C8	1.499 (2)
O4—C8	1.2034 (17)	C16—C17	1.388 (2)
O1—C7	1.3447 (17)	C16—H16	0.9300
O1—C1	1.4694 (16)	C14—C13	1.380 (2)
O3—C7	1.2101 (17)	C14—H14	0.9300
O6—N1	1.2283 (16)	C17—H17	0.9300
O7—N2	1.2267 (16)	C10—C11	1.385 (2)
O5—N1	1.2258 (16)	C10—H10	0.9300
O8—N2	1.2281 (17)	C20—C19	1.384 (2)
C9—C10	1.387 (2)	C20—H20	0.9300
C9—C14	1.393 (2)	C13—H13	0.9300
C9—C7	1.489 (2)	C4—C3	1.518 (2)
N1—C12	1.4710 (19)	C4—C5	1.526 (2)
N2—C18	1.4746 (18)	C4—H4A	0.9700
C18—C17	1.378 (2)	C4—H4B	0.9700
C18—C19	1.377 (2)	C11—H11	0.9300
C1—C6	1.510 (2)	C5—H5A	0.9700
C1—C2	1.5166 (19)	C5—H5B	0.9700
C1—H1	0.9800	C19—H19	0.9300
C12—C11	1.382 (2)	C3—C2	1.529 (2)
C12—C13	1.381 (2)	C3—H3A	0.9700
C6—C5	1.517 (2)	C3—H3B	0.9700
C6—H6	0.9800	C2—H2A	0.9700
C15—C20	1.385 (2)	C2—H2B	0.9700
			
C8—O2—C6	117.78 (12)	C9—C10—H10	119.8
C7—O1—C1	116.87 (11)	C15—C20—C19	120.09 (14)
C10—C9—C14	120.30 (13)	C15—C20—H20	120.0
C10—C9—C7	123.02 (13)	C19—C20—H20	120.0
C14—C9—C7	116.65 (13)	C14—C13—C12	118.21 (14)
O6—N1—O5	124.36 (13)	C14—C13—H13	120.9
O6—N1—C12	118.07 (13)	C12—C13—H13	120.9
O5—N1—C12	117.57 (13)	C3—C4—C5	110.47 (13)
O7—N2—O8	124.06 (13)	C3—C4—H4A	109.6
O7—N2—C18	118.23 (14)	C5—C4—H4A	109.6
O8—N2—C18	117.70 (14)	C3—C4—H4B	109.6
C17—C18—C19	123.34 (13)	C5—C4—H4B	109.6
C17—C18—N2	118.64 (14)	H4A—C4—H4B	108.1
C19—C18—N2	118.01 (13)	C12—C11—C10	117.91 (15)
O1—C1—C6	107.69 (12)	C12—C11—H11	121.0
O1—C1—C2	108.28 (11)	C10—C11—H11	121.0
C6—C1—C2	111.39 (12)	O3—C7—O1	124.61 (14)
O1—C1—H1	109.8	O3—C7—C9	122.21 (14)
C6—C1—H1	109.8	O1—C7—C9	113.17 (13)
C2—C1—H1	109.8	C6—C5—C4	110.17 (12)
C11—C12—C13	123.12 (14)	C6—C5—H5A	109.6
C11—C12—N1	118.90 (14)	C4—C5—H5A	109.6
C13—C12—N1	117.97 (13)	C6—C5—H5B	109.6
O2—C6—C1	105.67 (11)	C4—C5—H5B	109.6
O2—C6—C5	110.34 (12)	H5A—C5—H5B	108.1
C1—C6—C5	111.59 (13)	O4—C8—O2	124.52 (13)
O2—C6—H6	109.7	O4—C8—C15	124.49 (14)
C1—C6—H6	109.7	O2—C8—C15	110.98 (13)
C5—C6—H6	109.7	C18—C19—C20	118.17 (14)
C20—C15—C16	120.49 (14)	C18—C19—H19	120.9
C20—C15—C8	117.80 (14)	C20—C19—H19	120.9
C16—C15—C8	121.67 (14)	C4—C3—C2	110.83 (13)
C17—C16—C15	120.01 (14)	C4—C3—H3A	109.5
C17—C16—H16	120.0	C2—C3—H3A	109.5
C15—C16—H16	120.0	C4—C3—H3B	109.5
C13—C14—C9	120.12 (14)	C2—C3—H3B	109.5
C13—C14—H14	119.9	H3A—C3—H3B	108.1
C9—C14—H14	119.9	C1—C2—C3	110.46 (12)
C18—C17—C16	117.87 (14)	C1—C2—H2A	109.6
C18—C17—H17	121.1	C3—C2—H2A	109.6
C16—C17—H17	121.1	C1—C2—H2B	109.6
C11—C10—C9	120.32 (14)	C3—C2—H2B	109.6
C11—C10—H10	119.8	H2A—C2—H2B	108.1

### Hydrogen-bond geometry (Å, °)

**Table:**

*D*—H···*A*	*D*—H	H···*A*	*D*···*A*	*D*—H···*A*
C14—H14···O8^i^	0.93	2.40	3.240 (2)	150
C16—H16···O3	0.93	2.57	3.4374 (19)	156
C19—H19···O3^ii^	0.93	2.29	3.0919 (19)	145
C5—H5A···Cg2^iii^	0.97	2.79	3.7273 (18)	162

Symmetry codes: (i) −*x*, −*y*, −*z*+1; (ii) −*x*, *y*+1/2, −*z*+1/2; (iii) *x*, −*y*−1/2, *z*−3/2.
